# The Effect of Virtual Reality on Pain in Primiparity Women during Episiotomy Repair: A Randomize Clinical Trial

**Published:** 2015-05

**Authors:** Nahid JahaniShoorab, Samira Ebrahimzadeh Zagami, Ali Nahvi, Seyed Reza Mazluom, Nahid Golmakani, Mahdi Talebi, Ferial Pabarja

**Affiliations:** 1 Department of Midwifery, School of Nursing and Midwifery, Mashhad University of Medical Sciences, Mashhad, Iran;; 2 Faculty of Mechanical Engineering, K.N. Toosi University of Technology, Tehran, Iran;; 3 Psychiatry and Behavioral Sciences Research Center, Mashhad University of Medical Sciences, School of Medicine, Mashhad, Iran;; 4 The School of Nursing and Midwifery, Omolbanin Hospital, Mashhad, Iran

**Keywords:** Virtual reality, Pain, Parity, Episiotomy

## Abstract

**Background:**

Pain is one of the side effects of episiotomy. The virtual reality (VR) is a non-pharmacological method for pain relief. The purpose of this study was to determine the effect of using video glasses on pain reduction in primiparity women during episiotomy repair.

**Methods:**

This clinical trial was conducted on 30 primiparous parturient women having labor at Omolbanin Hospital (Mashhad, Iran) during May-July 2012. Samples during episiotomy repair were randomly divided into two equal groups. The intervention group received the usual treatment with VR (video glasses and local infiltration 5 ml solution of lidocaine 2%) and the control group only received local infiltration (5 ml solution of lidocaine 2%). Pain was measured using the Numeric Pain Rating Scale (0-100 scale) before, during and after the episiotomy repair. Data were analyzed using Fisher’s exact test, Chi-square, Mann-Whitney and repeated measures ANOVA tests by SPSS 11.5 software.

**Results:**

There were statistically significant differences between the pain score during episiotomy repair in both groups (P=0.038).

**Conclusion:**

Virtual reality is an effective complementary non-pharmacological method to reduce pain during episiotomy repair.

**Trial Registration Number: **IRCT138811063185N1.

## Introduction

Virtual Reality (VR) is a new technology^1^ by which a person in the virtual environment feels he/she is in the real world. This technology allows the user to interact with a computer (or other devices), that simulates the reality and the pain is reduced through diverting patient’s attention from the real world. It feels as if a person has become an active participant by visual, auditory, and other senses.^2^

During the past years, an effective use of virtual reality has been used to reduce pain in painful medical interventions, particularly on children, such as intravenous line insertion and even during chemotherapy in adults. Ramachandran and Rogers-Ramachandran used their first research in this area by building a virtual mirror in 1996. In 2000, Hoffman published the first valid results on this topic by using virtual reality as a method for pain relief during the burn wound dressing of two patients.^3^ Morris et al. in a study showed that virtual reality is significantly safe and beneficial in reducing pain.^4^

Episiotomy is a surgical procedure common in the delivery room.^5^ Medio-lateral episiotomy rate in 2009 was reported at 19% of vaginal deliveries and 40.6% in primiparity.^6^ Vakilian et al. reported episiotomy as routine in Iran for all primiparity women.^7^ However, this is now limited only to special cases.^8^

Pain is one of the side effects of episiotomy,^9^ and pain relief requested by a woman is considered as a medical indication for the use of pain relief methods. Nurses and midwives are responsible for responding to the need for pain relief (if needed).^10^ Poor management of pain, decreases the efficacy of therapeutic intervention.^11^ Jahani et al. reported a negative correlation between satisfaction and pain in the second stage of labor.^12^ Nowadays, the interest for using non-pharmacologic methods is increased due to the non-invasive nature and no severe side effects.^4^ The use of VR, as a non-invasive and analgesic method without drug addiction and minimum side effects is used in clinics.^2^ This study aims at determining the effect of virtual reality on pain in primiparity women during episiotomy repair.

## Materials and Methods

**Figure 1:**
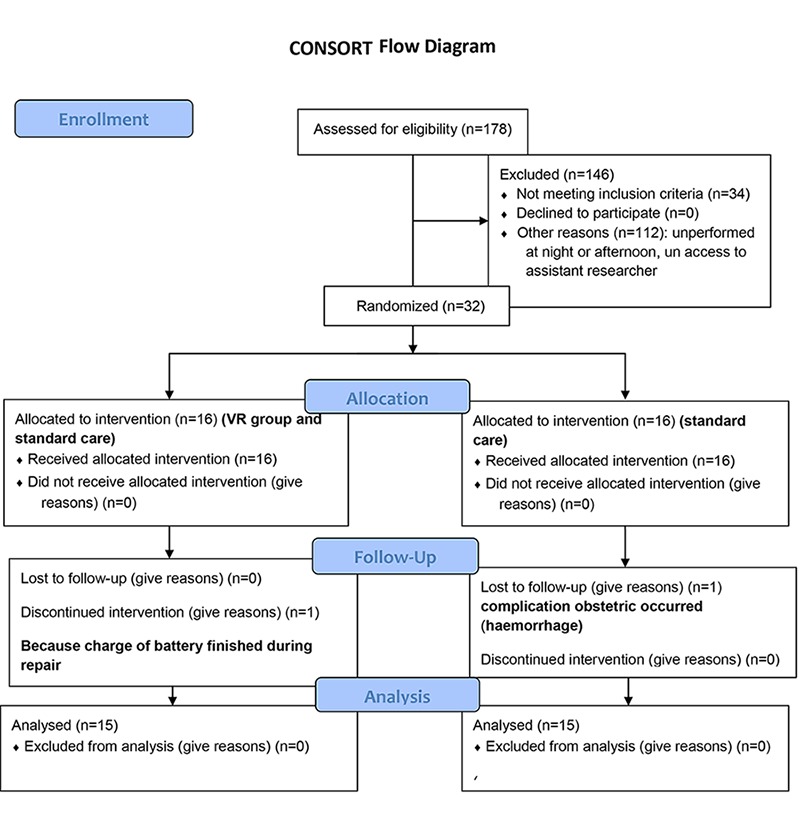
CONSORT diagram shows sampling procedures.

The sample size was estimated based on the results from a pilot study on 10 parturient women (power: 80%, confidence level: 95%). The estimation led to 13 parturient women; however, the sample size was increased to16 for a higher level of confidence in each group. Of the 178 primiparous referred to the Omolbanin Hospital of Mashhad during May to July 2012, 32 eligible women fulfilled the criteria and were selected for this study (figures 1). Women in the active phase of labor (dilation 4-5 cm) were randomly assigned into two groups (16 samples in each group). This study was approved by the Committee for Human Research at Mashhad Medical University with the registration NCT01659359. Informed written consent was obtained from each participant after full debriefing about the VR equipment.

The inclusion criteria were Iranian national, low risk of pregnancy without obstetric complication (hemorrhage, nonreassuring FHR) in all stages of labor, spontaneous labor associated episiotomy incision, no history of mental illness, addiction, motion sickness, and headaches. The exclusion criteria were Apgar-score <7 in 1 minute and 5 minutes of birth, neonate anomaly, and receiving unusual lidocaine during episiotomy repair (higher than 5 ml lidocaine 2%). The assistant researcher or assessor was an expert midwife with over twenty years of experience in this maternity unit. Initially, the assessor was trained by the researcher on video-glasses and the research methodology. All deliveries were done by the assistant researcher in lithotomy position. After delivery, the intervention group received VR added with anesthesia (video glasses and local infiltration 5 ml of lidocaine hydrochloride 2% solution) and the control group received standard anesthesia (local infiltration 5 ml of lidocaine hydrochloride 2% solution). In the VR group, prior to perineal repair, video glasses were deployed and the audio was adjusted to suit the patient. The repair method was the same in all patients and was done by the same expert midwife using a 2-0 chromic suture (SUPA, Iran). The length and depth of the wound were determined by a scaled sterile swab. 

The pain intensity was measured based on a Numeric Pain Rating Scale (0-100) before and during the four stages of repair, namely the first minutes of infiltration anesthesia during Hyman repair, during skin repair, immediately after the repair and the first hour after the repair. Data was collected using a questionnaire (including demographic, labor and delivery segments) and the VR equipment. After the episiotomy repair, the time was recorded from the first suture to the last. In addition, parturient satisfaction was recorded before and after episiotomy repair. The reliability of the questionnaire was assessed using the test-retest (r=0.84, CVI=0.75). 

Validity scores for NPRS and reliability were confirmed to be 0.76 and 0.90, respectively.^4^^,^^6^

Demographic information was analyzed with descriptive statistics including Mann-Whitney test for comparing pain score, episiotomy characteristics and clinical data and Fisher’s exact tests for baseline characteristics (age, education), pain intensity. In addition, analysis of repeated measures ANOVA was used for pain reduction efficacy between the five conditions (before and after). The data were analyzed by the SPSS 11.5 software.

*VR Equipment*

The VR equipment consisted of a video player (3D Blu-ray/DVD player full HD, model BD660, Indonesia) connected to a pair of video glasses (Wrap 920 system, Vuzix factory, USA) including the connection cables and one 3D film (IMAX Dolpine and Whales 3D 1080p). 

Video glasses include two miniature LCD viewing screens (for the right and left eyes) with 480×640 pixels resolution per display and two external headphones (stereo 60 Hz, 310 field view) weighing 85 grams. The unit included an external remote control device. 

## Results

Thirty-two patients were included in the study among which 30 patients were considered as eligible for data analysis. One patient was excluded due to malfunction of the remote control device (VR group) and the other patient due to delivery complication (control group).

The mean age of the patients was 24.1±4.1 (18-34 years) and 60% (n=18) had secondary or higher education level. The majority of patients in both groups were homemakers (93.3%, n=14). All patients were satisfied with the gender of their newborn. Mann-Whitney and Fisher’s tests did not show statistically significant differences between baseline characteristics (e.g. age, weight, education, employment status, satisfaction of pregnancy) and clinical data such as fatigue score or cervical dilation (table 1). Both groups were compared on oxytocin infusion in the first and second stage of labor and the extension of episiotomy to the vaginal walls (anterior, posterior, etc.) in the second stage of labor.

**Table 1:** Means±SD for the clinical data in two groups

**Variable**	**VR group**		**Non-VR group**		**Total**		**Mann-Whitney test**
	**Mean±SD**	**N**	**Mean±SD**	**N**	**Mean±SD**	**N**	
Fatigue scale (admit time)	36.6±33.7	15	27.2±26.1	15	31.9±30.0	30	U=96.0, P=0.480
Dilatation (cm) (admit time)	3.7±1.9	15	4.09±1.4	15	3.9±1.6	30	U=104.5, P=0.734
Duration of PROM (hour)	4.2±3.3	15	4.2±3.3	15	4.2±3.2	30	U=104.0, P=0.739
Duration of first stage labor (hour)	4.6±1.4	15	5.9±2.9	15	5.3±2.3	30	U=77.0, P=0.140
Duration of second stage labor (minute)	28±15.2	15	31±22.5	15	29±18.9	30	U=108.0, P=0.850

Fisher’s exact test showed no significant differences in any of the cases. The result showed significant differences on episiotomy incision depths between the intervention group and the group receiving standard care (U=69.0, P=0.042). Additionally, the same analyses were repeated with the inclusion of the duration of repair, and there was no significant difference between the groups (U=103.0, P=0.067). However, Mann-Whitney test showed patient with a VR distraction condition reported a significantly lower repair time than patients in non-VR group (table 2).

**Table 2:** Means±SD for episiotomy characteristics and duration of repair in two groups

**Variable**	**VR group**		**Non-VR group**		**Total**		**Mann-Whitney test**
	**Mean±SD**	**N**	**Mean±SD**	**N**	**Mean±SD**	**N**	
Episiotomy length (cm)	3.2±0.4	15	3.4±0.5	15	3.3±0.4	30	U=97.5, P=0.446
Episiotomy depth (cm)	2.5±0.6	15	2.1±0.5	15	2.3±0.6	30	U=69.0, P=0.042
Duration of repair (min)	11.4±2.6	15	13.6±3.3	15	12.5±3.2	30	U=103.0, P=0.067
Patient thinking about duration of repair (min)	6.8±2.4	15	13.3±8.9	15	9.6±7.1	30	U=55.0, P=0.013
Perineal body length	3.9±0.7	15	3.8±0.6	15	3.8±0.6	30	U=103.0, P=0.652

The pain score at different stages of the episiotomy in both groups was analyzed and compared by repeated measures ANOVA (table 3). We observed a significant difference between the groups, based group effect (P=0.038) and different stages (P<0.0001). The pattern of findings, as indicated by “group multiplied by the different stages of interaction effect”, was statistically significant for the pain intensity (group and stages P=0.044). Severe pain (from 80 to 100) was reported in 60% of the VR group and 20% of the non-VR group. Only 6.7% of the VR group and 26.7% of the non-VR group had severe pain on Hyman repair stage (table 4).

**Table 3:** Pain score (NRP: 0-100 mm) at different stages of the episiotomy in two groups

**Variable**	**VR group**		**Non-VR group**		**Total**	
	**Mean±SD**	**N**	**Mean±SD**	**N**	**Mean±SD**	**N**
Before repair	22.6±17.8	15	13.3±13.9	15	18.0±16.4	30
During of Hymen repair	9.0±12.6	15	23.6±19.8	15	16.3±17.9	30
During of Skin repair	16.7±16.5	15	39.3±22.5	15	28.0±22.5	30
After repair	6.0±12.8	15	25.2±14	15	10.0±20.1	30
In first hour	4.2±5.9	15	10.2±9.7	15	7.2±8.5	30
Repeated measures ANOVA	Main effect f=88.6, df=1, P<0.0001		Group f=4.8, df=1, P=0.038		Stages f=4.4, df=1, P=0.044	

**Table 4:** Pain intensity at different stages of the episiotomy in two groups

**Pain**		**VR group**	**Non-VR group**	**Total**	**P value**
		**N (%)**	**N (%)**	**N (%)**	
Before repair	No pain (0)	1 (6.7)	5 (33.3)	6 (20.0)	0.104
	Moderate (40-70)	10 (66.7)	9 (60.0)	19 (63.3)	
	Sever (80-100)	4 (26.7)	1 (6.7)	5 (16.7)	
During Hymen repair	No pain (0)	3 (20.0)	1 (6.7)	4 (13.3)	0.284
	Mild (10-30)	1 (6.7)	0 (0)	1 (3.3)	
	Moderate (40-70)	10 (66.7)	10 (66.7)	20 (66.7)	
	Sever (80-100)	1 (6.7)	4 (26.7)	5 (16.7)	
During skin repair	No pain (0)	3 (20.0)	1 (6.7)	4 (13.3)	0.076
	Moderate (40-70)	9 (60.0)	5 (33.3)	14 (46.7)	
	Sever (80-100)	3 (20.0)	9 (60.0)	12 (40.0)	
After repair	No pain (0)	9 (60.0)	6 (40.0)	15 (50.0)	0.524
	Moderate (40-70)	5 (33.3)	8 (53.3)	13 (43.3)	
	Sever (80-100)	1 (6.7)	1 (6.7)	2 (6.7)	
In first hour	No pain (0)	7 (46.7)	5 (33.3)	12 (40.0)	0.524
	Moderate (40-70)	8 (53.3)	10 (66.7)	18 (60.0)	

## Discussion

To our knowledge, this is the first use of VR in a maternity ward for pain relief in parturient women. The VR system used in this study was simple (lightweight, no head mount display and non-PC based) and appropriate for use in the delivery room. The results indicate that the clinical use of virtual reality (VR) with local anesthesia can reduce pain during the episiotomy repair more than those receiving standard care. These results concur with two other studies. Morris et al.^4^ reported that, approximately five samples (11 burn–injured patients) felt less severe pain during the usage of VR with analgesics condition than those in standard condition. Hoffman et al.^13^ found that all patients experienced significantly less pain (12 individuals). Wint et al. performed a pilot study on 30 cancer patients (10-19 years) and compared the efficacy of VR in puncture cerebrospinal fluid (CSF) in both VR and standard care conditions. The participants reported no significant difference in pain intensity.^14^

Based on Gate Control Theory of pain and previous experiences; parameters such as culture, stress and psychological factors have a powerful influence on the perception of pain by a patient and it effect pain signals perceived by the brain. The intensity of pain signals, depending on the patient’s concentration can be interpreted as very painful to mild pain.^13^ In the study by Wint et al., the cancer patients who were adolescents at the time of lumbar puncture, previously experienced lumbar puncture. However, in our study, the patients did not have prior experience with pain perception in episiotomy since they were primigravida.

Gershon et al. compared the condition caused by VR distraction, non-VR distraction, and standard care. The patients were children and adolescents (7-19 years) with cancer. The pain intensity was evaluated at the time of catheter insertion under the skin and the pain score was evaluated after joining the needle to catheter by a nurse. They found that all patients, especially those with higher age, experienced significantly less pain (P=0.03).^15^

Researchers have observed a difference related to the attractiveness of a film played by the VR system. The tendency of a patient to focus on a film influences the level of distraction caused by the VR technology and pain severity. Therefore, personal interest and preference of a patient should be considered when deploying a VR system.

In this study, similar to the study by Gershon et al., there was a wide age gap between the participants (18-34) and the patients selected one out of the five films presented to them (i.e. indicating common interest of the patients). One reason for the success of our research could be due to the appropriateness and attractiveness of the movies made available to the patients. Additional success factor was the integration of the parameter “culture” while selecting participants for this study. Note that in Gershon’s study, the patients were from different countries with different cultural backgrounds. Based on the Gate Control Theory, culture can affect pain perception. This clarifies our reasoning behind being an Iranian national as an inclusion criterion in the present study.

Patterson et al. were the first to use VR system to augment hypnosis.^2^^,^^16^ They confirmed the efficacy of VR on intensity reduction and unpleasantness of pain in a clinical case series of 21 patients who had been hospitalized due to acute trauma (P=0.04).

Recent studies suggest that not only the virtual environment in the path of the nerve pain interpretation makes a difference, but also reduce the perceived pain by decreasing brain activity on pain.^4^

Pain severity is another factor that should be considered when using virtual environments. McCaul and Malott announced that extreme stimulus prevents the effects of VR distraction.^17^ In other words; severe pain can obscure the beneficial effects of VR techniques.

In this study, based on NRPS, the mean of the pain score was <40. Sixty present of the non-VR group expressed severe pain during the skin repair, while the severe pain in the intervention group was 20% without statically significant difference (table 4)

Another interesting result from this study was a reduction in the perceived pain period during episiotomy. Patients stated that the perceived repair time was less than 46% of the actual time spent (P=0.013) (table2). 

Sharar et al. evaluated data from three studies and concluded that the combination of VR and standard analgesia reduced 20% pain intensity and 26% perceived pain period in 88 patients during post-burn physical therapy.^18^

In our study, there is a significant difference between the VR group and the non-VR group episiotomy incision depth (2.5±0.6 versus 2.3±0.6 cm, respectively) (P=0.042) in episiotomy repair labor, but there was not any statistically significant difference between the duration of repair in both groups. 

Similar to other studies, this study had certain limitations. The possibility of a single-blind design and the control of individual differences and previous experience of the patients were not possible. 

## Conclusion

Virtual reality is an effective complementary non-pharmacological method to reduce pain during episiotomy repair. 
